# Cocrystallization of Gefitinib Potentiate Single-Dose Oral Administration for Lung Tumor Eradication via Unbalancing the DNA Damage/Repair

**DOI:** 10.3390/pharmaceutics15122713

**Published:** 2023-11-30

**Authors:** Muhammad Inam, Yi Yang, Jialin Hu, Jiena Zheng, Wenxia Deng, You Zhou, Jialong Qi, Chuanshan Xu, Guihong Chai, Yuanye Dang, Wenjie Chen

**Affiliations:** 1 Key Laboratory of Molecular Target & Clinical Pharmacology and the State & NMPA Key Laboratory of Respiratory Disease, School of Pharmaceutical Science, The Fifth Affiliated Hospital, Guangzhou Medical University, Guangzhou 511436, China; 2022390020@gzhmu.edu.cn (M.I.); 2021210828@stu.gzhmu.edu.cn (Y.Y.); 2021210827@stu.gzhmu.edu.cn (J.H.); jienazheng@szmicro.onmicrosoft.com (J.Z.); d13535500519@163.com (W.D.); zyou16121@163.com (Y.Z.); xcshan68@gzhmu.edu.cn (C.X.); 2 Yunnan Digestive Endoscopy Clinical Medical Center, Department of Gastroenterology, The First People’s Hospital of Yunnan Province, Kunming 650032, China; qijialong1989@imbcams.com.ec; 3 School of Pharmaceutical Science, Sun Yat-sen University, Guangzhou 510006, China; chaigh@mail.sysu.edu.cn; 4 Sydney Vital Translational Cancer Research Centre, Westbourne St., Sydney, NSW 2065, Australia

**Keywords:** co-formers, gefitinib, cocrystal, DNA damage and repair, oral administration

## Abstract

Gefitinib (GEF) is a clinical medication for the treatment of lung cancer targeting the epidermal growth factor receptor (EGFR). However, its efficacy is remarkably limited by low solubility and dissolution rates. In this study, two cocrystals of GEF with co-formers were successfully synthesized using the recrystallization method characterized via Powder X-ray Diffraction, Fourier Transform Infrared Spectroscopy, and 2D Nuclear Overhauser Effect Spectroscopy. The solubility and dissolution rates of cocrystals were found to be two times higher than those of free GEF. In vitro cytotoxicity studies revealed that the cocrystals enhanced the inhibition of cell proliferation and apoptosis in A549 and H1299 cells compared to free GEF. In mouse models, GEF@TSBO demonstrated targeted, safe, and effective antitumor activity with only one-dose administration. Mechanistically, the GEF cocrystals were shown to increase the cellular levels of damaged DNA, while potentially downregulating PARP, thereby impairing the DNA repair machinery and leading to an imbalance between DNA damage and restoration. These findings suggest that the cocrystallization of GEF could serve as a promising adjunct to significantly enhance the physicochemical and biopharmaceutical performance for lung cancer treatment, providing a facial strategy to improve GEF anticancer efficiency with high bioavailability that can be orally administrated with only one dose.

## 1. Introduction

Solubility plays a pivotal role in the achieving of optimal drug absorption into the systemic circulation, ultimately facilitating the desired pharmacological response [1]. The poor solubility of drugs poses significant challenges in the development of formulations for both new chemical entities and generic drugs [2]. Remarkably, over 40% of pharmaceutical compounds exhibit either water insolubility or poor solubility. The indispensability of solubility in ensuring efficacious drug absorption is underscored by the imperative that the drug be in a soluble state at the site of absorption [3,4]. Various techniques can be employed to enhance the solubility of poorly soluble drugs, including physical and chemical modifications such as complexation, salt formation, particle size reduction, solid dispersion, and the use of surfactants [5]. Among the diverse routes of drug administration, oral drug delivery remains the preferred choice, predominantly owing to its convenience, ease of administration, high patient acceptability, and flexibility in dosage form design. Oral drug delivery is a commonly favored approach for drug administration through ingestion. It involves the passage of medication through the digestive system, enabling absorption into the bloodstream and distribution throughout the body. Patients prefer oral drug delivery due to its convenience, non-invasiveness, and ease of compliance. Some medications are better absorbed through the gastrointestinal tract, further solidifying oral administration as the preferred choice in many cases. As a result, many generic drug manufacturers have focused their efforts on the development of bioequivalent oral drugs [6,7]. Nevertheless, a substantial challenge within the domain of oral drug formulation pertains to the attainment of adequate bioavailability. Numerous factors contribute to drug bioavailability, encompassing aqueous solubility, dissolution rate, first-pass metabolism, presystemic metabolism, sensitivity to outflow mechanisms, and drug permeability. Poor solubility and limited permeability are common culprits, leading to reduced bioavailability [8,9]. Consequently, the enhancement of drug solubility and bioavailability persists as one of the most formidable challenges encountered in pharmaceutical advancement, particularly concerning oral drug delivery systems.

Pharmaceutical cocrystal is a crystalline material comprising an active pharmaceutical ingredient (API) and a co-former, forming solid-state associates bound by noncovalent interactions, such as hydrogen bonding, π-π stacking, or van der Waals forces [10,11]. Within a cocrystal, individual molecules maintain their distinct chemical identities while engaging in interactions that give rise to a novel material possessing altered physical characteristics, including enhanced solubility, stability, and bioavailability. Cocrystals hold promise in the realm of drug design, offering potential solutions to challenges encountered in drug development, particularly those associated with poor solubility, stability, and bioavailability [12]. The formation of cocrystals involving APIs and biologically active CFs, such as natural flavonoids, may present a means to attain improved safety profiles and enhanced therapeutic efficacy, offering a propitious avenue for achieving superior therapeutic attributes [13,14,15]. Currently, researchers are directing their efforts toward enhancing the solubility and dissolution rates of poorly water-soluble drugs. In comparison to alternative technologies such as osmotic pumps, film coating methods, and nanotechnology, cocrystallization technology has high flexibility and a simple production process [16,17].

Gefitinib (GEF) is an effective medication prescribed for patients with non-small cell lung cancer who exhibit resistance to chemotherapy. Its mechanism of action involves the selective inhibition of EGFR, a growth factor that regulates several critical cellular processes, including cell growth, apoptosis, and angiogenesis [18]. Upon administration, GEF undergoes extensive hepatic metabolism mediated by cytochrome P450 enzymes, primarily CYP3A4 and, to a lesser extent, CYP3A5 and CYP2D6 enzymes, resulting in the formation of five metabolites [19,20]. Pharmacokinetically, GEF is slowly absorbed after oral treatment, exhibiting a human bioavailability of approximately 60%. Owing to its high intestinal permeability and unfavorable physicochemical properties, such as low solubility and dissolution rate along the gastrointestinal tract, GEF falls under the category of class II biopharmaceutical drugs according to the Biopharmaceutical Classification System (BCS) [21,22]. Two cocrystals of GEF with 3-thiosemicarbano-butan-2-oneoxime (TSBO), a virus replication inhibitor [23], and Nicotinamide (NCA) [24] have been synthesized. These cocrystals, denoted as (GEF@TSBO) and (GEF@NCA), were evaluated for their saturated solubility and time-dependent dissolution rate. Furthermore, the anticancer effect of these cocrystals against A549 and H1299 lung cancer cells was investigated. In this work, TSBO and NCA were chosen as model co-formers owing to their proficiency as hydrogen-bonding donors and acceptors, notable solubility characteristics, and propensity for crystallization. The equilibrium between DNA damage and repair mechanisms is vital for maintaining genomic stability and preventing genetic mutations, including those linked to diseases such as cancer. Considering the mechanisms of unbalancing DNA damage and repair is crucial, especially in the context of disease development, therapy resistance, and personalized medicine. This understanding highlights the vital processes leading to genomic stability and identifies potential targets for therapeutic involvement. Certain substances, such as chemotherapeutic drugs or environmental agents, can hinder the repair machinery, leading to an accumulation of unrepaired DNA damage; however, the intrinsic cellular machinery of DNA damage repair plays a key role in sustaining the homeostasis of DNA damage and repair. In this work, GEF was used as the chemo drug, which has been reported to cause DNA damage and thus promote cell death, but the DNA damage repair pathway in cancer cells in turn largely decreases the level of damaged DNA, thus leading to less effect and even drug resistance [25]. In this regard, the incorporation of a co-former that may result in the inhibition of DNA damage repair could contribute to a loss of balance between DNA damage and the repair mechanism. In this manner, the cocrystallization of such a functional co-former with the API GEF would potentiate an enhanced antitumor effect. Herein, we evaluated lung tumor eradication by disturbing the delicate balance of DNA damage and repair mechanisms [26]. The GEF cocrystals have the capacity to complicatedly modulate the intricate DNA damage and repair processes essential to lung cancer cells. This modulation can ultimately lead to the elimination of lung tumors by interfering with critical DNA repair pathways vital for the survival of these cancerous cells. This scientific approach holds significant promise in advancing lung cancer therapy by precisely targeting the molecular mechanisms involved in DNA repair. These attributes collectively contribute to the enhanced performance of the active pharmaceutical ingredient (API) of GEF through cocrystal formulation.

## 2. Materials and Methods

### 2.1. Materials and Chemicals

Gefitinib (C_22_H_24_ClFNO_3_), Nicotinamide (C_6_H_6_N_2_O), Thiosemicarbazide (CH_5_N_3_S), and Diacetyl monoxime (C_4_H_7_NO_2_) with 99% purity (Figure S1) were purchased from Shanghai Macklin Biochemical Co. Ltd. (Shanghai, China). A549 human non-small cell lung cancer cell lines (Product No. SCSP-503) and H1299 human non-small cell lung cancer cell lines (Product No. SCSP-589) were purchased from Cell Bank, Chinese Academy of Sciences (Shanghai, China). The RPMI 1640 cell culture medium for lung cancer cells A549 and H1299 was purchased from Gibco Ltd. (Billings, MT, USA). All the other required materials, solvents, and chemicals were purchased from commercial sources, and used upon receiving.

### 2.2. Synthesis of TSBO

The TSBO molecule was synthesized according to the method previously reported [23] with slight modification, as shown in Figure S2. In brief, the mixture containing Thiosemicarbazide (0.45 g, 0.005 mol) and Diacetyl monoxime (0.51 g, 0.005 mol) was dissolved in a 50-mL MeOH-H_2_O mixture (1:1 *v*/*v*) and the solutions were stirred and refluxed for 3 h at 100 °C. Upon cooling, the solution was filtered and white crystalline-formed TSBO was obtained.

### 2.3. Fabrication of Cocrystals

The cocrystals were synthesized employing the recrystallization method [27]. In a round-bottom flask, an equimolar ratio of GEF (1 mmol, 446.9 mg) and TSBO (1 mmol, 174 mg) was combined and dissolved in a 10 mL mixture of EtOH-H_2_O (9:1 *v*/*v*). The solution was subjected to stirring at 90 °C and refluxed for 3 h. Upon completion of the reaction, the solution was allowed to cool to room temperature. Following a 72-h period, white crystalline GEF@TSBO cocrystals were obtained via a slow evaporation process. Similarly, an equimolar ratio of GEF (1 mmol, 446.9 mg) and NCA (122.12 mg) was dissolved in an EtOH-H_2_O mixture (7:3 *v*/*v*), and the solution was stirred and refluxed for 2 h at 65 °C. Subsequently, the solution was allowed to cool to room temperature, and after 48 h, white crystalline GEF@NCA cocrystals were obtained and collected for further analysis and experimentation.

### 2.4. General Characterizations

The powder X-ray diffraction (PXRD) patterns for the starting material and newly fabricated cocrystals were recorded on a Rigaku D/Max-2550PC, Tokyo, Japan. A rotating anode Cu-target X-ray with (λ = 1.5406) was used, worked at 40 kV, 250 mA, with a range of scanning of 3.0 to 90° with a 5° per min speed, and an increasing step size of 0.02° in a time of 0.5–3 s. Fourier transform infrared spectroscopy (FT-IR) spectra were recorded with a Thermo Scientific Nicolet iS50 FT-IR spectrometer (Thermo Fisher Scientific Co., Ltd. Waltham, MA, USA) in the spectral range of 400–4000 cm^−1^ in the potassium bromide (KBr) diffuse reflectance mode. About 2 mg of the sample with 100 mg of KBr was manually mixed in a mortar and pressed into thin pellets. Further data were analyzed using spectrum software GraphPad Prism 6.0. The 2D-NOESYspectrum was recorded on a Bruker DRX-400 spectrometer. GEF@TSBO (15 mg) and GEF@NCA (15 mg) were separately dissolved in DMSO-d_6_, samples were examined at room temperature, and spectra were analyzed with MestReNova 14.3.1 software. The morphology of the cocrystal was tested by scanning electron microscope (SEM) (Sigma 500, ZEISS, Oberkochen, Germany). The bulk samples were photographed at an accelerating voltage of 4 kV without any coating. A TA DSCQ100 differential scanning calorimeter (TA Instruments, New Castle, Germany) was used for the DSC analysis. It was heated at a rate of 10 °C/min with a nitrogen flow of 50 cm^3^/min. The powder samples utilized for differential scanning calorimeter (DSC) studies weighed between 4 to 7 milligrams. A 35–500 °C temperature range was scanned and ±0.02 °C was the calibrated temperature accuracy.

### 2.5. Physicochemical Evaluation

Solubility and dissolution studies of GEF, GEF@TSBO, and GEF@NCA were performed using a Thermo Logical Advancement UV-vis spectrometer. These experiments were conducted in distilled water with a pH of 7.0 at a controlled temperature of 37 °C. To initiate the experiments, the solutions were deliberately supersaturated and maintained under constant stirring at 150 rpm using a magnetic stirrer for a period of 24 h at the specified temperature. Afterward, the suspension was filtered through a Whatman’s 0.45 mm syringe channel, accordingly diluted, and the concentration was determined using a standard curve (Figure S3), which was plotted by measuring the absorbance of different concentrations at 330 nm (λ_max_). For the dissolution rate assessment, the samples were introduced into 200 mL of water and subjected to stirring at 150 rpm for a duration of 3 h, maintaining the temperature at 37 °C. At predefined intervals, 3 mL of the disintegration medium was withdrawn, and an equivalent volume of fresh medium was added to ensure a consistent volume. The absorbance at λ_max_ was recorded for each of the withdrawn solutions.

### 2.6. Molecular Docking

Autodock 4.0 software was employed for conducting docking calculations, aimed at optimizing the probable geometric crystal structure of the cocrystal [6]. Subsequently, molecular docking was performed to evaluate the binding of the cocrystal with the active site of the protein receptor, focusing on ligand-protein binding efficiency [28]. The protein receptor site EGFR crystal file (pdb ID: 1xkk, Uniport name: EGFR-Human) was downloaded from the protein data bank. The supposed optimized structure of the cocrystal was simulated and subjected to docking with the protein receptor, utilizing Autodock 4.0. The binding free energy within the target receptor was computed for the most favorable binding pose. Visual representations of the best-docked poses were obtained using Autodock. The best-docked complexes were carefully chosen from docking based on the binding free energy value and Discovery Studio visualizer (http://accelrys.com/products/collaborative-science/biovia-disco very-studio (accessed on 7 July 2023)) was used to assess molecular interactions.

### 2.7. In Vitro Cytotoxicity

The primary objective of the present study was to evaluate the in vitro cytotoxicity of GEF, GEF@NCA, and GEF@TSBO in A549 and H1299 lung cancer cell lines, employing the CCK-8 assay, a well-established method for evaluating cellular viability [29]. In 96-well plates, a total of 5 × 10^3^ cells/well of A549 and H1299 were seeded and allowed to incubate for a duration of 24 h. Subsequently, varying concentrations of GEF ranging from (0 to 150 μM), GEF@NCA (0 to 45 μM), and GEF@TSBO (0 to 70 μM), suspended in culture medium, were introduced to replace the existing medium. Following an additional incubation period of 24 h, a 10% CCK-8 solution (GlpBio, Montclair, CA, USA) was added to each well, and the absorbance was measured at 450 nm using an Epoch microplate reader (BioTek, Winooski, VT, USA). Finally, cell viability was calculated using the following formula. Overall, this experiment was designed to assess the potential antitumor effects against lung cancer cell lines and provide insights into their possible clinical applications.

$$Cell\;viability\left(\%\right)=\frac{\left[A(dosing)-A\left(blank\right)\right]}{\left[A\left(control\right)-A\left(blank\right)\right]}\times 100\%$$

A(dosing): Absorbance of wells with cells, CCK-8 solution, and drug treatments.
A(blank): Absorbance of wells with medium and CCK-8 solution without cells.
A(control): Absorbance of wells with cells, CCK-8 solution without drug treatments.

### 2.8. Cellular Uptake

The intracellular distribution of GEF, GEF@NCA, and GEF@TSBO in A549 and H1299 cells was investigated through CytoFLEX flow cytometry. To facilitate this examination, 1 × 10^6^ cells per well of A549 and H1299 were seeded in six-well plates and allowed to incubate for a duration of 24 h. Subsequently, GEF, GEF@NCA, and GEF@TSBO (each at 50 μM) were introduced and further incubated for 12 h. After the incubation period, the cells were washed with PBS, trypsinized, and transferred to a flow cytometer. The obtained data were analyzed using FlowJo software v10 from the United States.

### 2.9. Cell Apoptosis Assay

The present study aimed to investigate the effect of GEF, GEF@NCA, and GEF@TSBO on cell apoptosis using annexin V-FITC/PI kit. GEF was used as a probe, as it has its own probe (ex 405 nm, em 525 nm). To achieve this objective, A549 and H1299 lung cancer cells were seeded in six-well plates at a density of 1 × 10^6^ cells/well and treated with 50 μM of the compounds for 24 h. After treatment, the cells were collected and washed with PBS buffer and stained with an annexin V-FITC/PI apoptosis assay kit. The percentage of apoptotic cells was measured using CytoFLEX flow cytometry.

### 2.10. Cell Clonogenic Assay

A cell clonogenic assay was conducted to assess the impact of GEF, GEF@NCA, and GEF@TSBO on cell proliferation. In six-well plates, A549 and H1299 cells were initially seeded at a density of 250 cells per well and allowed to incubate for 24 h. Subsequently, these cells were exposed to a 50 μM concentration of the compounds for an additional 24 h. The culture medium containing the respective drug was replaced with fresh medium, and the cells were permitted to proliferate for a period spanning 14 days. The experiment was considered concluded when the control group exhibited the formation of more than 50 cell colonies. The cells were then fixed, stained using crystal violet, and photographed for statistical analysis. This experimental design aimed to evaluate the potential anti-proliferative effects of these compounds in lung cancer cell lines, which could provide valuable insights into their therapeutic potential for cancer treatment.

### 2.11. Western Blot

A549 and H1299 cells were cultured in six-well plates with a seeding density of 1 × 10^6^ cells/well for 24 h. Subsequently, the cells were treated with 50 μM of GEF, GEF@NCA, or GEF@TSBO for an additional 24 h. After treatment, the cells were harvested and lysed using protease inhibitors on ice for 30 min. The lysate was then centrifuged at 12,000 rpm for 10 min at 4 °C, and the supernatant was quantified with a BCA Protein Assay Kit (Yeasen Bio., Shanghai, China). Proteins were separated on a 10% Bis-Tris polyacrylamide gel (Beyotime, Nantong, China) and then transferred onto a PVDF membrane (BioRad, Hercules, CA, USA). The membrane was blocked with 5% skim milk powder (Beyotime, Nantong, China) and incubated with primary antibodies against GAPDH (Servicebio, Wuhan, China), γ-H2AX (CST, Danvers, MA, USA), and PARP (CST, USA) overnight. After an hour of incubation, protein expression levels were detected using ECL chemiluminescence (Beyotime, Nantong, China) with a suitable secondary antibody (Yeasen Biotechnology, Shanghai, China).

### 2.12. Antitumor Effect In Vivo

The in vivo antitumor efficacy of GEF@TSBO cocrystal was assessed utilizing a subcutaneous xenograft human lung cancer cell model. To initiate the study, approximately 1×10^7^ A549 cells were injected into the right leg of female Balb/C nude mice. The experimental protocol was initiated once the tumor volume reached approximately 100 mm^3^. Various pharmaceutical formulations were either intravenously administered (i.v.) through the tail vein or intragastrically administered (i.g.), adhering to the experimental schedule. The experimental animals were systematically randomized into four distinct groups: a control group treated with PBS, Group 2 who received an i.v. injection of GEF, Group 3 subjected to an i.v. injection of a mixture of GEF and TSBO, and Group 4 administered with an i.v. injection of cocrystal GEF@TSBO. Additionally, Group 5 was designated for the i.g. administration of cocrystal GEF@TSBO. The dose of each group was normalized to GEF is 25 mg/kg. Throughout the course of the study, the length (L) and width (W) of the tumors were measured utilizing a Vernier caliper, and tumor volumes were computed using the formula V_tumor_ = 0.5 × L × W^2^. Body weight, a vital parameter for characterizing GEF@TSBO toxicity, was also meticulously recorded. Upon the culmination of the study, all experimental mice were humanely euthanized, and their tumors and major organs were meticulously harvested. Subsequently, these tumors and organs were preserved in formalin for immunohistochemical assays, conducted in accordance with the approvals from the Animal Ethics Committee of Guangzhou Medical University.

### 2.13. Statistical Analysis

To determine statistically significant differences between groups in all experiments, paired *t*-tests (for between) and one-way analysis of variance (ANOVA) were used. All data are expressed as the mean of standard deviation (SD), with a *p* < 0.05 considered statistically significant.

## 3. Results and Discussion

**Figure 1:**
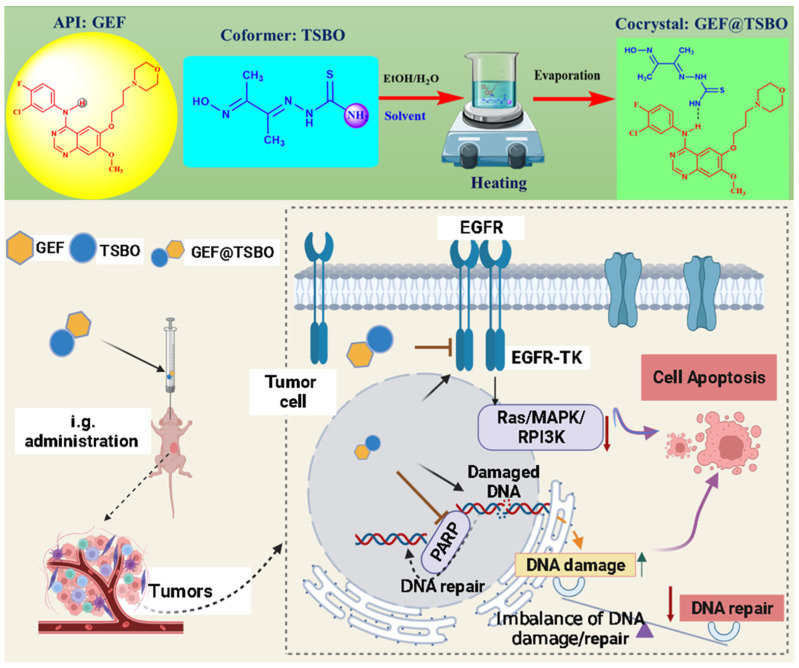
The schematic illustration represents (**top**) the formulation of cocrystal by the noncovalent hydrogen bonding interaction between API (GEF, the EGFR-TK inhibitor) and (**bottom**) co-former in crystal lattice and oral drug administration, leading to the imbalance of DNA damage and repair and inhibiting of cancer cell growth by increased cell apoptosis.

Pharmaceutical cocrystal formulation has emerged as a topic of significant interest due to its potential to alter the physicochemical and biopharmaceutical attributes of active pharmaceutical ingredients (APIs) while preserving their therapeutic efficacy. This innovative approach holds promise for the advancement of oral drug delivery methods by facilitating administration in a crystalline state [30]. Cocrystals exhibit a range of advantageous features, including low toxicity, protective characteristics, drug delivery capabilities, and the potential to enhance the therapeutic effects of APIs [16]. The current emphasis on cocrystal formulation stems from its capacity to enhance the biopharmaceutical properties (Figure 1) of drugs by accumulating therapeutically active constituents without necessitating chemical modifications [31]. In this study, Gefitinib (GEF), categorized as a BCS class II drug owing to its limited solubility and high permeability, served as the subject of investigation. Two novel pharmaceutical cocrystals of GEF in association with TSBO and NCA were meticulously prepared, yielding notable improvements in both physicochemical and biopharmaceutical attributes. As a preliminary step towards obtaining the crystalline cocrystal form, the recrystallization method was employed. Subsequently, a diverse array of characterization techniques was deployed to scrutinize the solid-phase cocrystal formulation in this study.

### 3.1. Synthesis and Characterization

**Figure 2:**
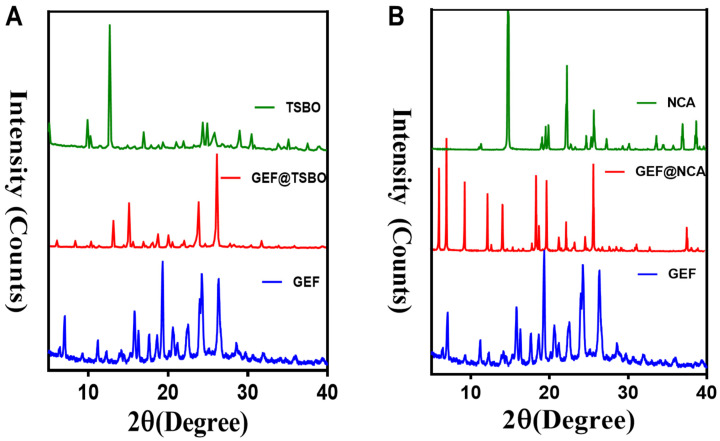
Represents the P-XRD pattern comparison of (**A**) GEF@TSBO and (**B**) GEF@NCA with the respective material of the cocrystal.

The cocrystals GEF@NCA and GEF@TSBO were prepared in crystalline form (Figure S4) using equivalent molar ratios in a mixture of ethanol and water under different precipitation temperatures. Various characterization techniques were employed to analyze the cocrystals. Power X-ray diffraction (PXRD) is a reliable analytical technique for crystallographic material characterization, and changes in crystallinity patterns can indicate the successful formulation of a new crystalline phase [32]. PXRD analysis confirmed the formation of the new cocrystal phases. The cocrystal GEF@TSBO exhibited characteristic peak values at 2θ (7.13°, 9.40°, 11.30°, 14.10°, 15.9°, 17.80°, 18.61°, 19.50°, 20.7022.61°, 24.53°, and 26.8°), while the cocrystal GEF@NCA exhibited characteristic peaks at 2θ (6.17°, 7.10°, 9.55°, 12.40°, 14.2°, 18.20°, 18.40°, 18.80°, 19.7°, 21.10°, 22.30°, 23.4°, 24.80°, and 25.90°). Additionally, many characteristic peaks of the free GEF and its corresponding co-former shifted in the new crystalline cocrystal phase, indicating the formation of the cocrystal (Figure 2A,B).

Cocrystal formation is based on the establishment of hydrogen bonding interactions API and the co-former, demonstrating apparent shifts in functional group frequencies. These shifts are readily determined through Fourier-transform infrared (FT-IR) analysis [33]. The FT-IR analysis of the cocrystals revealed the following: the free GEF exhibited a characteristic medium peak at 1561 cm^−1^ corresponding to the NH group of the amine. A shift in the NH stretching frequency was observed in the GEF@TSBO cocrystal to 1579 cm^−1^. Similarly, TSBO exhibited a medium peak at 3324 cm^−1^ corresponding to the NH2 group of the amide. A change in frequency was observed in the GEF@TSBO cocrystal to 3346 cm^−1^. These changes in functional group frequencies indicated weak hydrogen bonding (NH…NH_2_), confirming (Figure S5A) the formation of the GEF@TSBO cocrystal. The free GEF exhibited a strong and broad peak at 775 cm^−1^ corresponding to the amine group. A shift in frequency was observed in the GEF@NCA cocrystal to 790 cm^−1^. Similarly, NCA exhibited a medium peak at 3152 cm^−1^ corresponding to NH2. In the GEF@NCA cocrystal, the frequency shifted to 3260 cm^−1^. These frequency shifts indicated strong hydrogen bonding in the functional groups of GEF and NCA (NH…NH2), confirming (Figure S5B) the formulation of the new crystalline GEF@NCA cocrystal. The NOESY experiment is a useful tool to determine relations between protons and provide information about the configuration of molecules [34]. The 2D-NOESY method has been used to investigate and identify the interaction groups, as well as to measure the interaction strength associated between API and the co-former that form the cocrystal [6].

The 2D-NOESY analysis of the cocrystals is presented in Figure S6A,B. This analysis revealed a correlation between the NH group of GEF at 7.69 ppm and the NH_2_ group of TSBO at 7.75 ppm in the GEF@TSBO cocrystal, as well as a correlation with the NH_2_ group of NCA at 7.79 ppm, indicating an interaction between them in the cocrystal. Figure 2 clearly demonstrates that the interaction between GEF and NCA is more pronounced compared to TSBO, suggesting a stronger interaction in the GEF@NCA cocrystal.

DSC analyses are widely used for the determination of thermal stability. The DSC analysis of cocrystals is shown in Figure S7A. The GEF@TSBO thermal curve indicates an exothermic peak at 187 °C due to a phase transition/rearrangement and a crystalline, sharper endothermal peak at 214 °C with no weight loss in TGA (Figure S7B). The compound is thermally stable up to 214 °C, and it thermally decomposes in two successive mass loss phases that match exothermic events in the DSC curves. Similarly, GEF@NCA shows an endothermal peak at 194 °C with no weight loss. The only existing endothermic peak, due to the melting of the cocrystal, confirms the GEF@NCA crystal to be an anhydrous form.

### 3.2. Solubility and Dissolution Rate

The impact of solid forms of APIs on solubility and the time-dependent dissolution rate of drugs is widely recognized [35]. Therefore, selecting the appropriate form of the active pharmaceutical ingredient (API) is crucial for successful drug formulation [36]. Gefitinib (GEF) is categorized as a Class II drug within the Biopharmaceutical Classification System (BCS) due to its limited aqueous solubility, a characteristic that consistently leads to reduced bioavailability [21,22]. The comprehensive solubility results for GEF and its cocrystals are depicted in Figure S8A. Notably, the cocrystals of GEF with TSBO and NCA exhibited higher solubility compared to free GEF. Moreover, the dissolution rate of the cocrystal was also higher than that of GEF, as illustrated in Figure S8B. This exceptional solubility and time-dependent dissolution rate of the cocrystal can be attributed to the presence of rich hydrogen bonding interactions, which enable more competitive interactions with the solution media and facilitate the dissociation of the cocrystal’s subcomponents. Consequently, the formulation of GEF cocrystals successfully enhances the solubility and dissolution rate of GEF, potentially improving its bioavailability and therapeutic effect.

### 3.3. Molecular Docking Study

Molecular docking stands as an indispensable tool for forecasting and comprehending the intricate interactions between multiple molecules, ultimately culminating in the establishment of stable crystal structures within cocrystals. This method holds paramount significance in cocrystal design by facilitating the selection of prospective cocrystal partners. Molecular docking, at its core, involves the simulation of binding among multiple molecules, predicting their energetics and the geometry of their interactions. This process often relies on computational software to meticulously compute the optimal molecular orientation, energy considerations, and geometric characteristics necessary to attain the stability of cocrystals. In the sphere of pharmaceutical formulation development, molecular docking plays a pivotal role, particularly as it enables the amalgamation of multiple components to optimize drug properties. The burgeoning interest in cocrystals as a means to enhance drug efficacy and stability has accentuated the significance of molecular docking in this realm [37].

**Figure 3:**
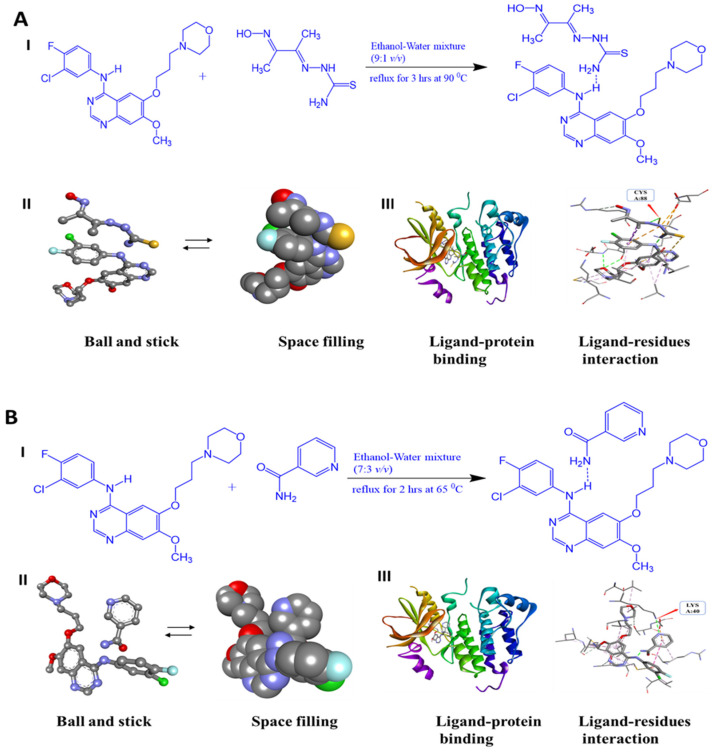
Molecular docking study of GEF cocrystals: (**I**) Chem draw illustration highlighting noncovalent hydrogen bonding between the GEF and co-former; (**II**) Optimized geometric crystal structure with space filling and ball and stick; (**III**) Cocrystal binding mode analysis of top poses in 1xkk protein active sites showing ligand-protein binding and ligand-residue interaction of (**A**) GEF@TSBO and (**B**) GEF@NCA cocrystal.

As shown in Figure 3, the illustration showcases the most probable geometric structure optimization of cocrystals, conscientiously determined using Autodock 4.0 computational techniques. The docking process takes into account a multitude of energy-related parameters, encompassing computed docking models, geometric conformation, complementarity scores, sizes of interface areas, attractive and repulsive forces, atomic content, and energies, along with the contribution of hydrogen bonding to the cocrystal formation [38]. Furthermore, the molecular docking study serves the purpose of evaluating the predictive therapeutic efficiency of the cocrystal. Within this context, the molecular docking analysis of the cocrystal with the EGFR receptor forecasts the ligand’s (cocrystal) binding conformation to the specific target (protein) binding site. Despite the generation of multiple docking poses through the process, only the docking pose characterized by minimal energy interaction between the protein and cocrystals is considered, thereby ensuring the precise alignment of the ligand within the receptor. Consequently, this results in the stabilization of the docked ligand with minimal energies of −9.62 kcal/mol (GEF@TSBO) and −9.59 kcal/mol (GEF@NCA). Notably, the molecular docking interactions of GEF@TSBO reveal vigorous conventional hydrogen bonding interactions between TSBO and the CYS A:88 residue of the target protein. Similarly, in the case of GEF@NCA, the NCA molecule is predicted to exhibit formidable conventional hydrogen bonding interactions with the LYS: A40 residue of the target protein. The potent interactions between the cocrystals and amino acids facilitate their deep penetration into the receptor protein cavity, serving as anticancer agents to inhibit cancer cell growth.

### 3.4. In Vitro Cytotoxic Effect

**Figure 4:**
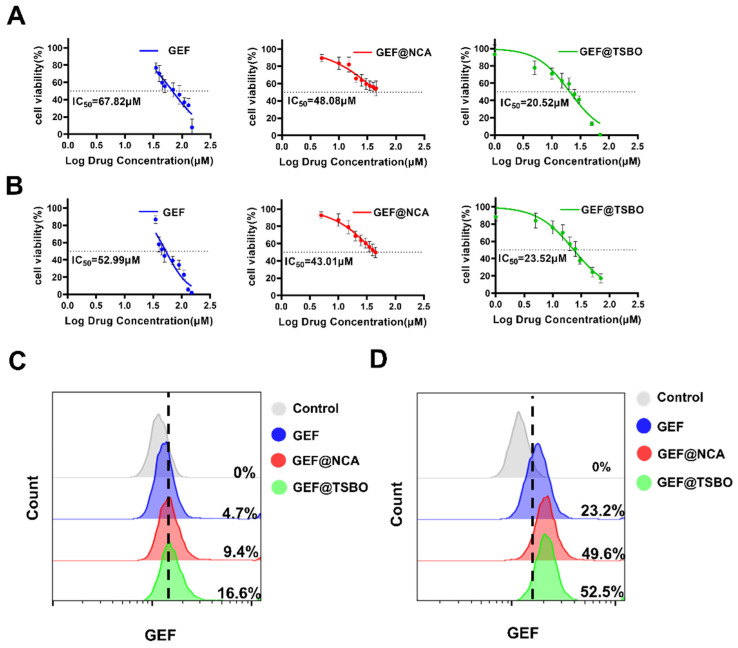
In vitro cytotoxic effect and cellular uptake. (**A**) Cytotoxic effects of GEF, GEF@NCA, and GEF@TSBO on lung cancer cells A549 and (**B**) H1299 in vitro at different concentrations. (**C**) Quantitative uptake of GEF, GEF@NCA, and GEF@TSBO by A549 and (**D**) H1299 cells using flow cytometry.

For the assessment of cytotoxicity, A549 and H1299 cells were subjected to varying concentrations of GEF, GEF@NCA, and GEF@TSBO over a 24-h incubation period. The results are presented in Figure 4A,B. Free GEF exhibited slight toxicity towards A549 and H1299 cells due to the higher concentration required to achieve a 50% reduction in cell viability. In contrast, GEF@NCA and GEF@TSBO demonstrated stronger cytotoxicity that was dependent on the dose used. For A549 cells, the IC50 (half-maximal inhibitory concentration) after 24 h of incubation was 67.82 μM for free GEF, 48.08 μM for GEF@NCA, and 20.52 μM for GEF@TSBO. Similarly, for H1299 cells, the IC50 values were 52.99 μM for GEF, 43.01 μM for GEF@NCA, and 23.52 μM for GEF@TSBO. It is noteworthy that the cocrystals exhibited the lowest IC50 values, indicating higher cytotoxicity compared to free GEF. Furthermore, free GEF showed some inhibitory effect on the growth of A549 and H1299 cells compared to the control group, although this effect was not significant. This may be attributed to its poor solubility. Conversely, the cocrystals had a more pronounced inhibitory effect on the growth of A549 and H1299 cells within the same time frame (as shown in Figure S9). This enhanced effect may be due to the presence of NCA and TSBO eutectic drugs, which could promote the uptake of the cocrystals by tumor cells [39]. Notably, GEF@TSBO displayed a significant decrease in cell viability compared to both free GEF and the GEF@NCA cocrystal. Most remarkably, GEF@TSBO exhibited an obvious decrease in cell viability compared to both free GEF and the GEF@NCA cocrystal. This intriguing result emphasizes the potential of TSBO to enhance drug absorption and bear higher anticancer efficacy against cancer cells, thereby possibly concluding in enhanced biopharmaceutical performance.

### 3.5. Cellular Uptake of Cocrystals

Flow cytometry was employed for a quantitative assessment of the cellular uptake of GEF, GEF@NCA, and GEF@TSBO in tumor cells. In A549 cells, notable variations in mean fluorescence intensities were observed between GEF and cocrystals. Specifically, the cocrystals GEF@NCA and GEF@TSBO exhibited enhanced cellular uptake (Figure 4C). Similarly, the uptake of GEF@NCA and GEF@TSBO in H1299 cells was significantly higher compared to the GEF pure drug group (*p* < 0.001) (Figure 4D). These findings indicate that GEF@NCA and GEF@TSBO are readily absorbed by tumor cells and can exert more potent inhibitory effects on cell growth compared to free GEF.

### 3.6. Cell Apoptosis Assay

**Figure 5:**
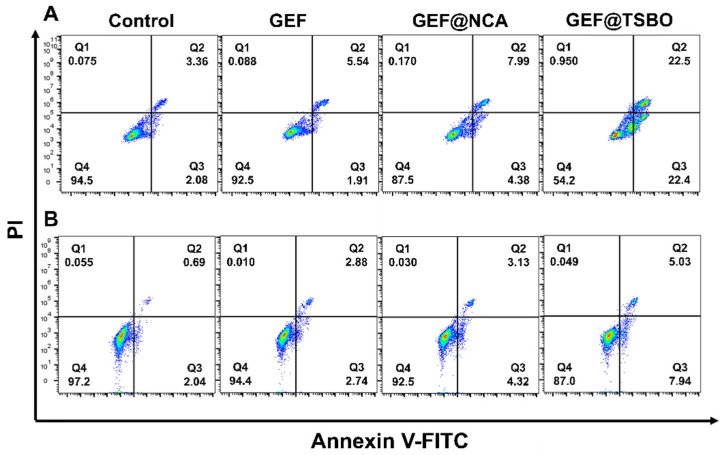
In vitro apoptosis assessment of A549 cells (**A**) and H1299 cells (**B**) treated with GEF, GEF@NCA, and GEF@TSBO.

To assess the efficacy of free GEF, the cocrystal GEF@NCA, and GEF@TSBO, A549 and H1299 cells were co-incubated with these samples. The results are shown in Figure 5. Subsequent to a 24-h incubation period, flow cytometry was employed for the quantification of apoptosis rates. Under the observed concentrations, free GEF exhibited minimal cytotoxicity, with apoptosis rates (Q2 + Q3) registering at a mere 7.45% for A549 cells and 5.62% for H1299 cells. In contrast, GEF@NCA displayed an elevated apoptosis rate (Q2 + Q3) of 12.37% for A549 cells, while H1299 cells exhibited a rate of 7.45%. Notably, the apoptosis rates of GEF@TSBO (Q2 + Q3) were significantly augmented, measuring 44.9% for A549 cells and 12.97% for H1299 cells, surpassing those of both free GEF and GEF@NCA. These results are in concordance with the observations made in the cell viability assays, reaffirming the heightened potency of GEF@TSBO.

### 3.7. Cell Clonogenic Assay

**Figure 6:**
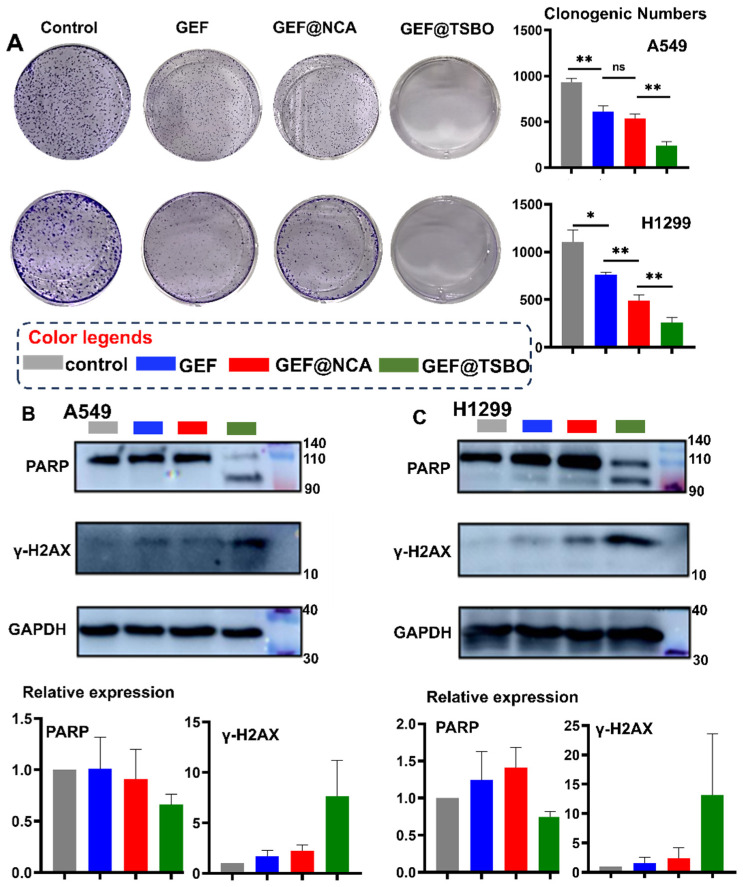
(**A**) Cell clonogenic assay of different treatments in A549 cells and H1299 cells. Western blot analysis and correlated quantitation (bottom bar charts) of protein PAPR and γ-H2AX in (**B**) A549 cells and (**C**) H1299 cells treated with GEF, GEF@TSBO, and GEF@NCA after incubation. GAPDH was used as a control. Statistical significances between every two groups were calculated via paired student *t*-test. * *p* < 0.05, ** *p* < 0.01, ns stands for no-significance.

The cell clonogenic assay is an in vitro method used to assess the ability of a single cell to form colonies. Figure 6A illustrates the results, indicating that the control generated approximately 1000 colonies. In contrast, GEF treatment resulted in only around 500 colonies, which is similar to the colony count observed with GEF@NCA. Interestingly, no colonies formed when cells were treated with GEF@TSBO. These findings demonstrate that GEF@TSBO significantly inhibits colony formation in A549 and H1299 cells, displaying a stronger effect compared to the other cocrystal, GEF@NCA.

### 3.8. Western Blotting Analysis

To assess the influence of GEF cocrystals on the expression of pivotal proteins associated with DNA damage and repair pathways, Western blot analysis was conducted (Figure 6B,C). In both A549 and H1299 cells subjected to GEF@NCA and GEF@TSBO treatment, a notable increase in the expression of the DNA damage marker γ-H2AX was evident, in stark contrast to the control and free GEF-treated cells. Conversely, the DNA damage repair protein marker PARP displayed downregulation in both A549 and H1299 cell lines. These results suggest that GEF@TSBO has the potential to increase the number of apoptotic cells by inducing imbalanced and damaged DNA levels. This effect is attributed to the increased cellular DNA damage and the inhibition of DNA damage repair processes. Importantly, GEF@TSBO exhibited a stronger impact on DNA damage and the inhibition of the DNA repair factor PARP when compared to other treatments [40,41].

### 3.9. Antitumor Effect In Vivo

**Figure 7:**
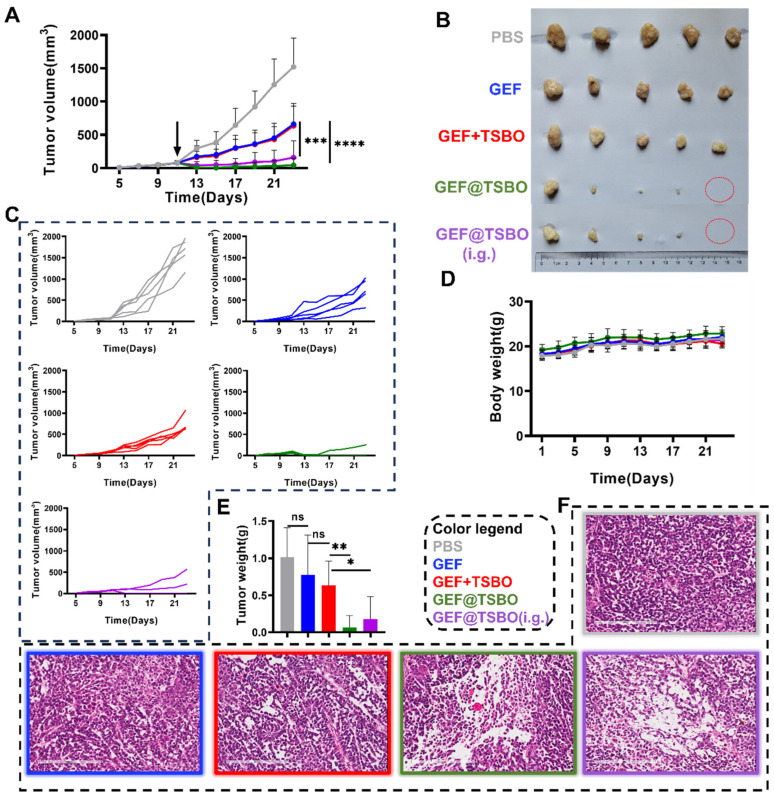
The studies of anticancer effect in vivo. (**A**) Tumor volume of mice after one dose of treatment, the arrow indicates dose administration at day 11. (**B**) Tumor photographs obtained from BALB/c-nuc mice bearing A549 of different groups after treatment, red circles mean those tumors eradicated at the endpoint. (**C**) Tumor growth curves in each group. (**D**) Body weight of mice throughout treatment. (**E**) Weight of mouse tumors. (**F**) H&E staining image of tumor after treatment. Scale: 200 μm. Data are expressed as standard deviations ± averages. Statistical significances between every two groups were calculated via paired student *t*-test. * *p* < 0.05, ** *p* < 0.01, *** *p* < 0.001, **** *p* < 0.0001, ns stands for no-significance.

**Figure 8:**
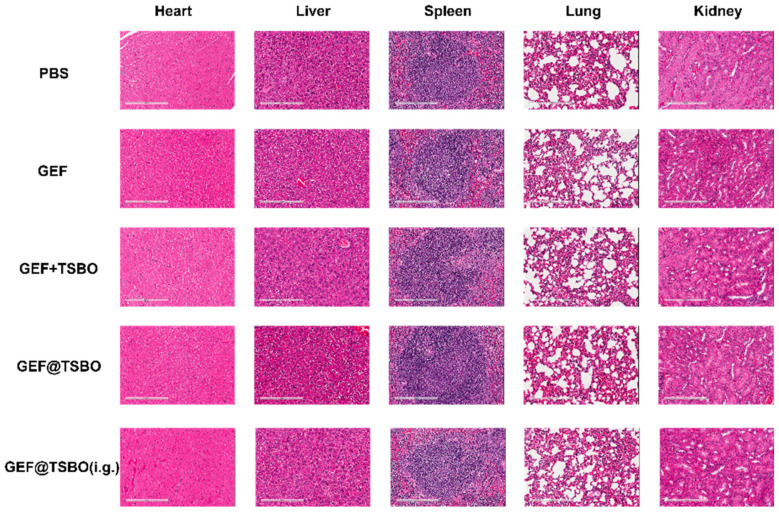
H&E staining of major organs (heart, liver, spleen, lungs, and kidneys). Scale bars: 200 μm.

GEF@TSBO was chosen as the cocrystal drug for mice treatment based on its superior ability to induce DNA damage, as demonstrated in in vitro tests. To investigate the viability of the GEF@TSBO cocrystal drug via oral administration, intragastric (i.g.) administration was concurrently conducted. The dose of each group normalized to GEF was 25 mg/kg. Notably, the tumor volume in the GEF@TSBO i.v. group approached zero, even surpassing the original volume (Figure 7A), with complete tumor eradication observed in two instances (Figure 7B). The tumor growth curves (Figure 7C) for each group clearly illustrate the tumor inhibition trends following drug treatments. Similarly, the i.g. group, receiving the same GEF@TSBO dose, also exhibited significant tumor regression compared to the GEF + TSBO mixture group, with a tumor growth curve closely resembling that of the i.v. GEF@TSBO group. These findings validate the effective elimination of tumors and highlight the superiority of the GEF@TSBO cocrystal compared to the physical mixture of GEF and TSBO, possibly attributed to the enhanced physicochemical and biopharmaceutical properties of the cocrystal (Figure 7E). Notably, the orally administered group (i.g.) also exhibited substantial tumor inhibition, with the complete eradication of the tumor, indicating the superior bioavailability and therapeutic potential of the orally administered cocrystal drug formulation. The size and weight of the spleen corresponded to the tumor size (Figure S10A,B), indicating the absence of significant immunotoxicity. Moreover, the mice in different groups did not exhibit substantial weight loss throughout the experiment, signifying good biocompatibility and an absence of acute toxicity for GEF@TSBO (Figure 7D). Examination of tumor sections stained with hematoxylin and eosin (H&E) (Figure 7F) revealed that mice treated with GEF@TSBO exhibited the most pronounced pathological damage in tumor tissue, featuring larger necrotic areas compared to other treatments. These histological findings corroborate the in vitro results, highlighting the considerable therapeutic potential of the combination therapy. Various blood routine parameters and blood biochemical parameters, including ALT (alanine aminotransferase), AST (aspartate aminotransferase), BUN (blood urea nitrogen), CR (creatinine), and CK (creatine kinase), remained within normal ranges compared to mice treated with PBS (Figure S11). Collectively, these findings indicate that GEF@TSBO presents no significant biosafety concerns when juxtaposed with GEF and extends its potential for in vivo applications. Additionally, H&E staining of vital organs, including the heart, liver, spleen, lungs, and kidneys (Figure 8), displayed no significant differences between the groups, consistent with previous results.

## 4. Conclusions

Cocrystal synthesis allows for the formation of functional materials and systems through the association of molecular building blocks in a non-covalent manner. In the field of pharmaceuticals, cocrystallization has become a popular approach for improving the clinical efficacy of orally administered drugs. Cocrystals have the ability to impact drug physicochemical and various aspects of drug pharmacokinetics. This study elaborates on the cocrystal formation of GEF with TSBO and NCA through a recrystallization technique. GEF solubility and dissolution rate were substantially improved as a result of cocrystallization. The resulting cocrystal showed notably improved physicochemical properties and anticancer effects with only one-dose oral administration. Additionally, the cocrystals proved more effective in inhibiting the growth of A549 and H1299 cells compared to free GEF. The cocrystals were demonstrated to accumulate intracellular levels of DNA damage through the downregulation of the DNA damage repair regulator PARP. This resulting inhibition of PARP expression significantly compromises the efficacy of the DNA repair apparatus, establishing an imbalance tipped towards heightened DNA damage over effective restoration processes. Based on the promising pharmacokinetics, the antitumor effects on the tumor model revealed that GEF@TSBO significantly enhanced tumor eradication.

## Data Availability

The datasets created throughout this work are available upon request.

## Supplementary Materials

The following supporting information can be downloaded at: https://www.mdpi.com/article/10.3390/pharmaceutics15122713/s1, Figure S1. The chemical structure of (A) Thiosemicarbazide, (B) Diacetyl monoxime, (C) Nicotinamide (NCA), (D) Gefitinib (GEF) and (E,F) the simulated and experimental Powder XRD comparison of GEF and NCA; Figure S2. Proposed mechanism of the synthesis of TSBO molecule; Figure S3. Represents the standard curve of (a) Pristine GEF (b) cocrystal GEF@TSBO, (c) cocrystal GEF@NCA, and (d) the UV absorbance spectra at λ max. Figure S4. SEM images of (A) GEF@NCA and (B) GEF@TSBO. Figure S5. FT-IR comparison of (A) GEF@TSBO and (B) GEF@NCA with their respective components. Figure S6. The 2D NOESY of (A) GEF@NCA and (B) GEF@TSBO. Figure S7. (A) DSC and (B) TGA of GEF@TSBO and GEF@NCA. Figure S8. Evaluation of physicochemical characteristics where (A) represents the saturation solubility and (B) time-dependent dissolution rate of pristine GEF, cocrystal GEF@TSBO and GEF@NCA in pH 7.0 water. Figure S9. Cell viability of (A) A549 and (B) H1299 against GEF, GEF@NCA and GEF@TSBO. Figure S10. (A) Weight of mouse spleen. (B) Spleen images obtained from BALB/c-nuc mice carrying A549 after treatment. Figure S11. In vivo systemic toxicity assay after administration GEF@TSBO. Variations of ALT (Alanine aminotransferase), AST (Aspartate aminotransferase), BUN (Blood urea nitrogen), CR (creatinine), CK (Creatine Kinase). Table S1. In vivo systemic toxicity assay after administration GEF@TSBO. WBC (White blood cell), Lymph (Lymphocyte), Mon (Monocytes), Gran (Granulocyte).
