# Supplementary material for: Cocrystallization of Gefitinib Potentiate Single-Dose Oral Administration for Lung Tumor Eradication via Unbalancing the DNA Damage/Repair
Source: Pharmaceutics. 2023 Nov 30;15(12):2713. doi: 10.3390/pharmaceutics15122713 (PMC10747925; doi:10.3390/pharmaceutics15122713)
Supplement: Supplementary file 1 [file pharmaceutics-15-02713-s001.zip › pharmaceutics-2690723-supplementary.pdf]

pharmaceutics

MDPI

Supplementary Materials: Cocrystallization of Gefitinib Potentiate Single-Dose Oral Administration for Lung Tumor Eradication via Unbalancing the DNA Damage/Repair

Muhammad Inam, Yi Yang, Jialin Hu, Jiena Zheng, Wenxia Deng, You Zhou, Jialong Qi, Chuanshan Xu, Guihong Chai, Yuanye Dang and Wenjie Chen

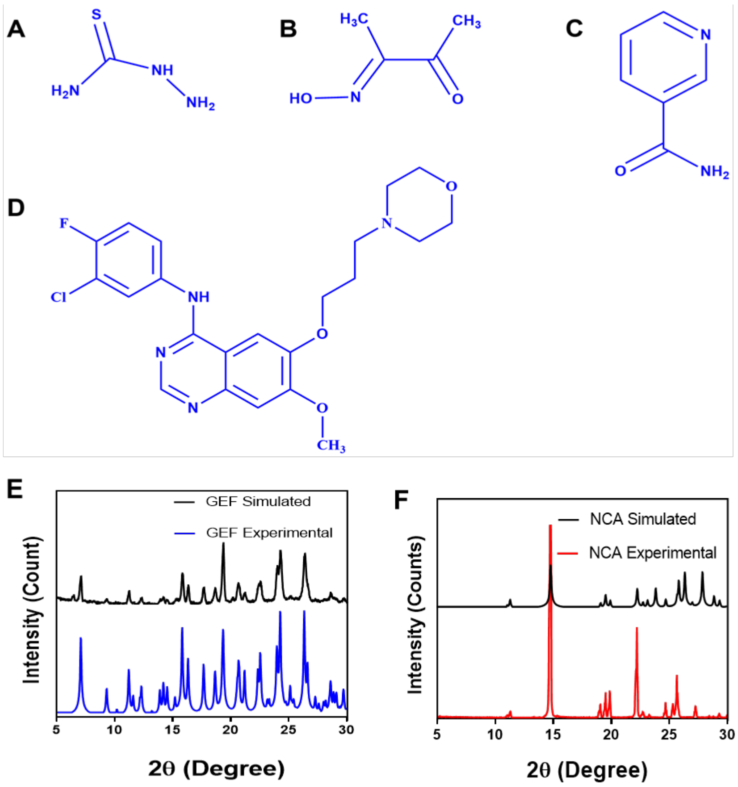

Figure S1. The chemical structure of (A)Thiosemicarbazide, (B) Diacetyl monoxime, (C) Nicotinamide (NCA), (D) Gefitinib (GEF) and (E, F) the simulated and experimental Powder XRD comparison of GEF and NCA.

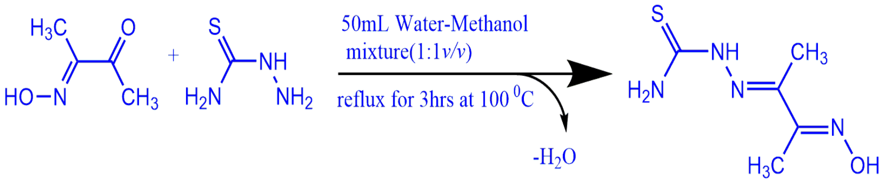

Figure S2. Proposed mechanism of the synthesis of TSBO molecule.

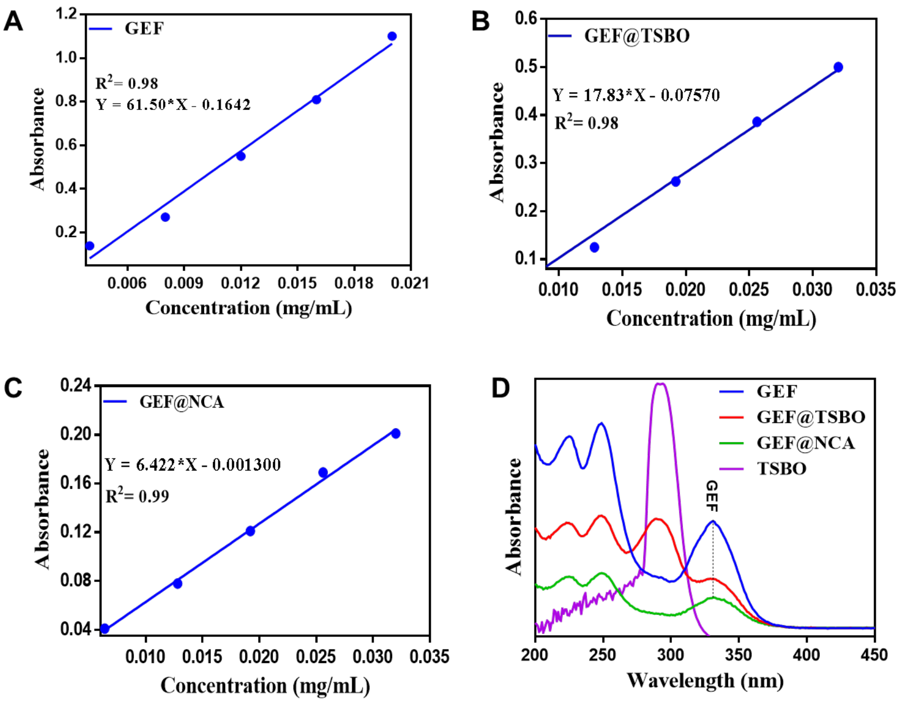

Figure S3. Represents the standard curve of (a) Pristine GEF (b) cocrystal GEF@TSBO, (c) cocrystal GEF@NCA, and (d) the UV absorbance spectra at λ max.

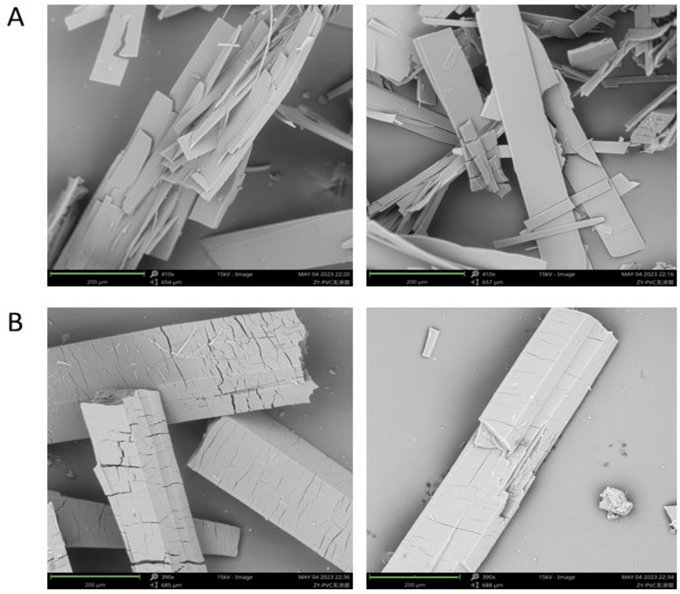

Figure S4. SEM images of (A) GEF@NCA and (B) GEF@TSBO.

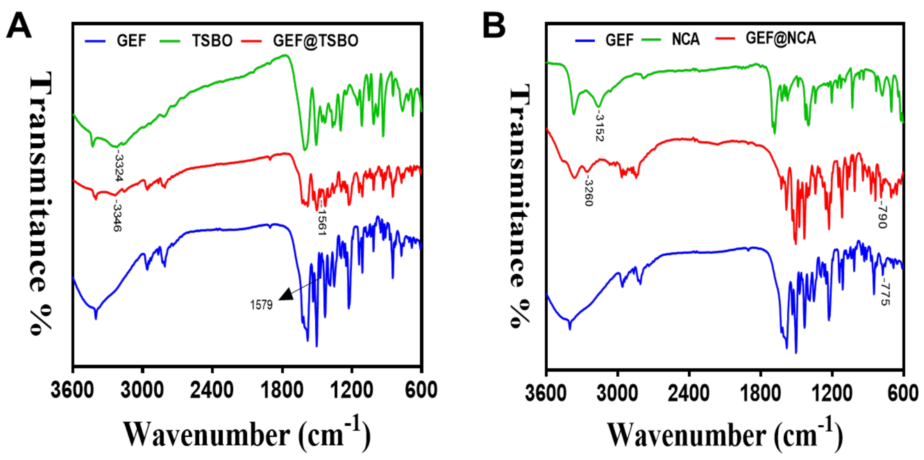

Figure S5. FT-IR comparison of (A) GEF@TSBO and (B) GEF@NCA with their respective components.

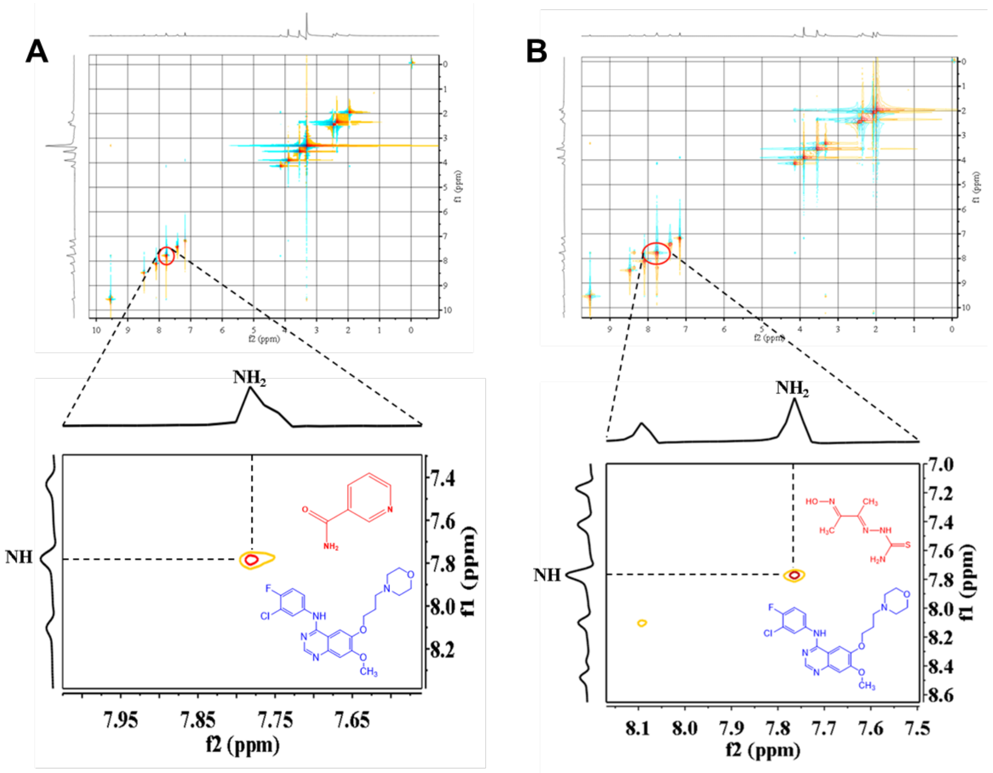

Figure S6. The 2D NOESY of (A) GEF@NCA and (B) GEF@TSBO.

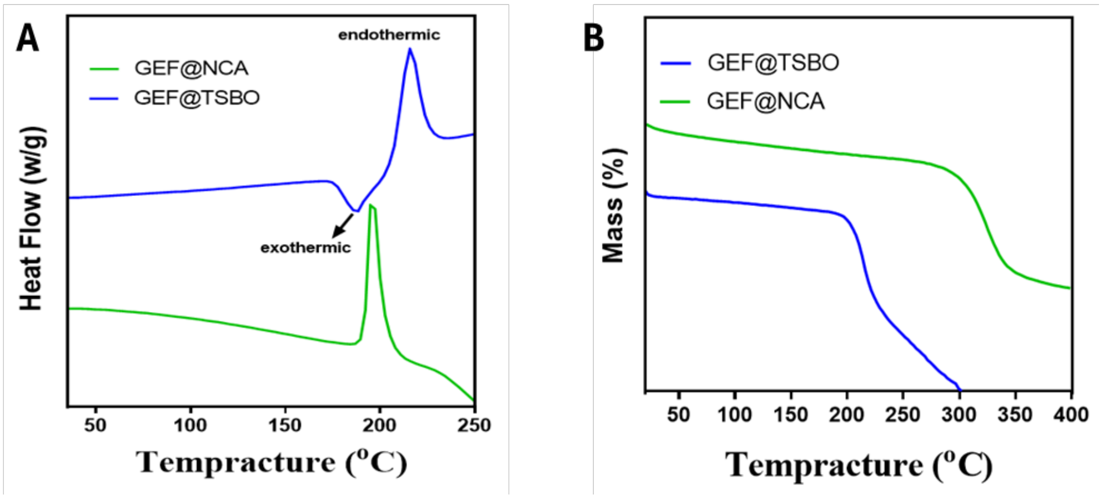

Figure S7. (A) DSC and (B) TGA of GEF@TSBO and GEF@NCA.

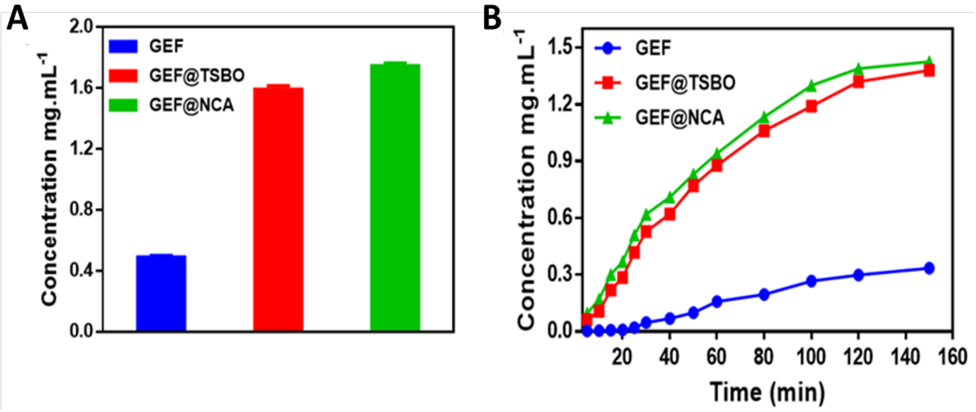

Figure S8. Evaluation of physicochemical characteristics where (A) represents the saturation solubility and (B) time-dependent dissolution rate of pristine GEF, cocrystal GEF@TSBO and GEF@NCA in pH 7.0 water.

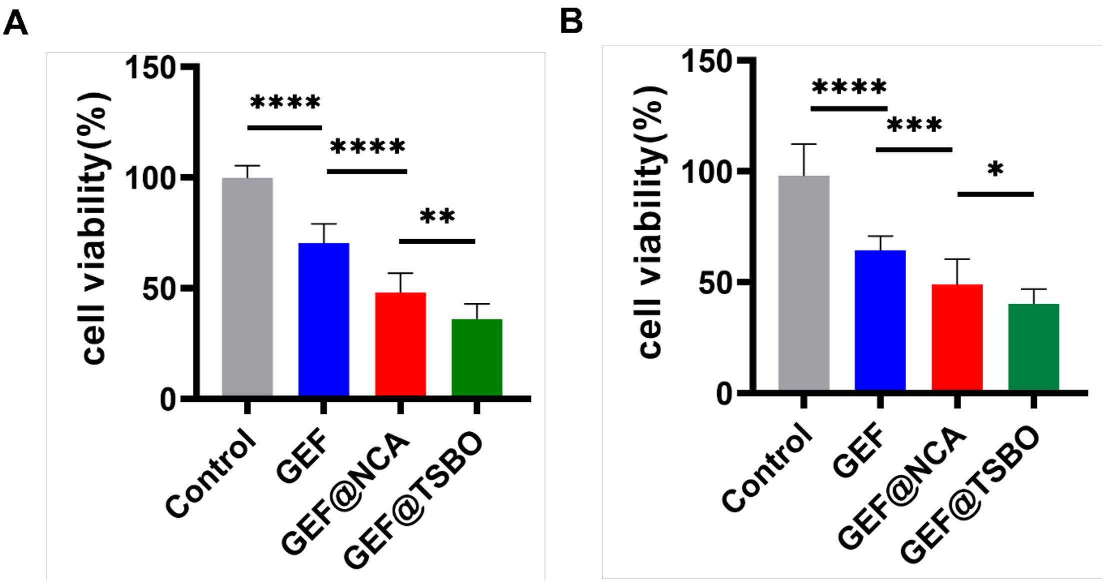

Figure S9. Cell viability of (A) A549 and (B) H1299 against GEF, GEF@NCA and GEF@TSBO.

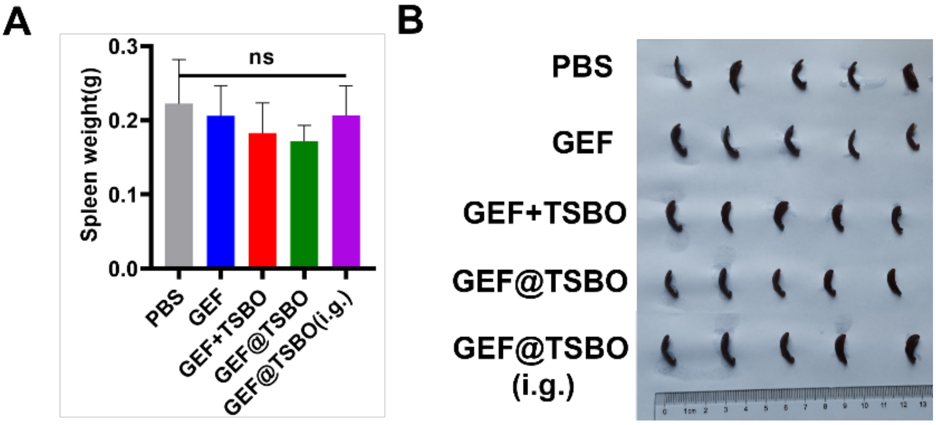

Figure S10. (A) Weight of mouse spleen. (B)Spleen images obtained from BALB/c-nuc mice carrying A549 after treatment.

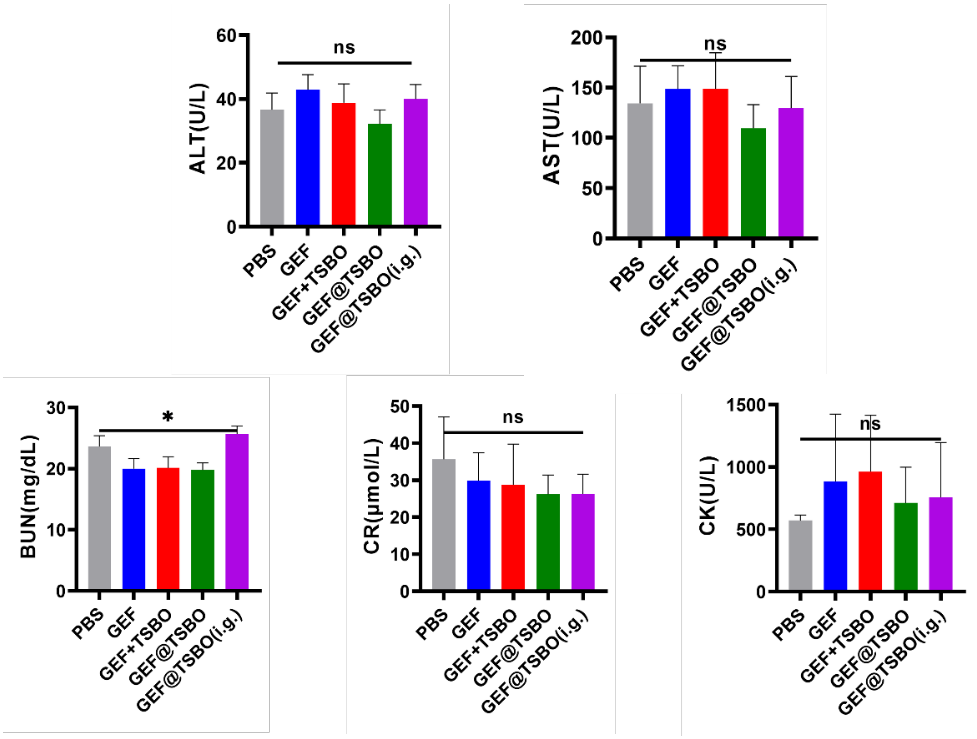

Figure S11. In vivo systemic toxicity assay after administration GEF@TSBO. Variations of ALT (Alanine aminotransferase), AST (Aspartate aminotransferase), BUN (Blood urea nitrogen), CR (creatinine), CK (Creatine Kinase).

Table S1. In vivo systemic toxicity assay after administration GEF@TSBO. WBC (White blood cell), Lymph (Lymphocyte), Mon (Monocytes), Gran (Granulocyte).

Group	Parameter	Outcome	Unit
**PBS**	WBS	4.5	10^9/L
	Lymph	3.9	10^9/L
	Mon	0.1	10^9/L
	Gran	0.5	10^9/L
**GEF**	WBS	8.1	10^9/L
	Lymph	8.4	10^9/L
	Mon	4	10^9/L
	Gran	1	10^9/L

**GEF+TSBO**	WBS	3.4	10^9/L
	Lymph	2.2	10^9/L
	Mon	0.2	10^9/L
	Gran	1.3	10^9/L
**GEF@TSBO**	WBS	4	10^9/L
	Lymph	2.5	10^9/L
	Mon	0.2	10^9/L
	Gran	1.3	10^9/L
**GEF@TSBO (i.g.)**	WBS	5	10^9/L
	Lymph	3.2	10^9/L
	Mon	0.2	10^9/L
	Gran	1.6	10^9/L
